# Leveraging the human microbiota to target bacterial respiratory pathogens: new paths toward an expanded antimicrobial armamentarium

**DOI:** 10.1128/mbio.00854-23

**Published:** 2023-06-20

**Authors:** Jillian H. Hurst, Matthew S. Kelly

**Affiliations:** 1 Department of Pediatrics, Division of Infectious Diseases, Duke University School of Medicine, Durham, North Carolina, USA; 2 Duke Microbiome Center, Duke University School of Medicine, Durham, North Carolina, USA; University of Connecticut, Storrs, Connecticut, USA

**Keywords:** *Moraxella catarrhalis*, antibiotic resistance, human microbiome, respiratory pathogens, comparative genomics, *Rothia *species, microbial ecology, acute otitis media

## Abstract

Acute respiratory infections are the most frequent infections across the lifespan and are the leading infectious cause of death among children globally. Bacterial respiratory infections are routinely treated with antibiotics, nearly all of which are derived from microbial natural products. Unfortunately, antibiotic-resistant bacteria are an increasingly frequent cause of respiratory infections, and there are few new antibiotics in development that target these pathogens. In the article by Stubbendieck et al., the authors identified *Rothia* species that demonstrate *in vitro* and *ex vivo* growth inhibition of the respiratory pathobiont *Moraxella catarrhalis*. The authors present experiments suggesting that this activity is mediated at least in part through the secretion of a novel peptidoglycan endopeptidase that targets the *M. catarrhalis* cell wall. In this commentary, we discuss these findings in the context of the urgent threat of antimicrobial resistance and highlight the promise of the human respiratory microbiota as a source of novel biotherapeutics.

Acute respiratory infections are the most common infections at all ages and are associated with substantial morbidity and mortality. Globally, these infections account for nearly four million deaths each year, most of which result from pneumonia and occur in low- and middle-income countries (LMICs) (1). Other respiratory infections, including acute otitis media (AOM) and acute bacterial rhinosinusitis, are rarely life-threatening but have an enormous societal burden. For example, more than five million cases of AOM occur each year among children in the United States resulting in direct health expenditures exceeding $6 billion (2). The development and deployment of vaccines against *Streptococcus pneumoniae* and *Haemophilus influenzae* type b have been a cornerstone of recent public health efforts to reduce the global burden of respiratory infections. However, these vaccines are only effective in preventing infection caused by vaccine serotypes, and vaccine introduction has been followed by the emergence of non-vaccine serotype strains that are highly virulent and multidrug resistant. Additionally, while these vaccines have been associated with a reduction in respiratory infections such as AOM, the proportion of these infections caused by *M. catarrhalis* and other pathobionts for which there is no licensed vaccine has increased (3, 4).

*M. catarrhalis* is a Gram-negative, nonencapsulated diplococcus that colonizes the upper respiratory tracts of 30%–100% of infants and 1%–5% of adults. Although the majority of individuals colonized by *M. catarrhalis* are asymptomatic, this species is a leading cause of AOM, chronic obstructive pulmonary disease (COPD) exacerbations, and bacterial rhinosinusitis (5). There are currently no licensed vaccines for *M. catarrhalis*; moreover, a leading vaccine candidate was recently found to be ineffective in preventing moderate or severe COPD exacerbations, although efficacy studies have not been conducted in other conditions or among children (6). Antibiotic resistance is also a significant concern in treating infections caused by *M. catarrhalis*. Approximately 90% of strains are resistant to amoxicillin, the first-line antibiotic for respiratory infections among children, with increasing resistance to macrolides and fluoroquinolones (7). Considering the substantial role of this species in respiratory infections and the negative consequences of the use of broad-spectrum antibiotics, effective vaccines and new therapeutics are urgently needed for *M. catarrhalis*.

In the article by Stubbendieck and colleagues (8), the authors found that the abundance of the bacterial genus *Rothia* was negatively correlated with *M. catarrhalis* carriage in a small cohort of children. After cultivating *Rothia* strains from nasal samples from these children, the authors screened these strains for inhibition of *M. catarrhalis* growth. They then used a combination of comparative genomics and proteomics to identify potential mechanisms underlying this activity, leading ultimately to the identification and purification of a putative endopeptidase, SagA, which was demonstrated to degrade *M. catarrhalis* peptidoglycan. Finally, the authors showed that strains of two *Rothia* species, *Rothia aeria* and a potential novel species provisionally named *R. similmucilaginosa*, reduce colonization of human respiratory epithelial cells by *M. catarrhalis*. Here, we seek to provide context to these findings and their importance to efforts to combat antimicrobial resistance and develop novel therapeutics to prevent or treat respiratory infections.

The overuse and misuse of antibiotics in humans and livestock and increased human mobility have fueled the rapid expansion and global dissemination of antibiotic resistance determinants. In the United States alone, antimicrobial-resistant organisms cause more than 2.8 million infections and 35,000 deaths each year (9). Over the past decade, notable progress has been made in slowing the spread of some antimicrobial-resistant organisms; however, antibiotic resistance to several common bacterial respiratory pathogens rose substantially during this time period. In particular, increasing resistance of *S. pneumoniae* to beta-lactam antibiotics and macrolides was observed globally, with the prevalence of multidrug-resistant (non-susceptible to ≥3 antibiotic classes) and extensively drug-resistant (non-susceptible to ≥5 antibiotic classes) strains in some regions approaching 50% and 20%, respectively (10). Similarly, several countries reported a high and rising prevalence of both multidrug-resistant and extensively drug-resistant *H. influenzae* strains (11, 12). Although antibiotic resistance affects all countries, the burden is higher in the world’s poorest nations. Nearly 90% of deaths attributable to bacterial antibiotic resistance, including more than 99.6% of child deaths, occur in LMICs (13). A 2016 analysis estimated that antibiotic resistance will result in more than 10 million deaths annually by 2050, underscoring the critical need for development of new antibiotics (14).

Clinically relevant bacteria have developed substantial resistance to most existing classes of antibiotics, making identification of new antibiotic classes vital to addressing the growing threat of antimicrobial resistance. Between 1930 and 1962—often referred to as “the golden age of antibiotics”—more than 20 classes of antibiotics were brought to market in the United States (15). Unfortunately, these early successes in antibiotic development led to the widespread belief that antibiotics would eliminate infectious diseases which, when combined with the high costs of research and development, discouraged pharmaceutical companies from further investments in the antibiotic pipeline. As a result, only two new antibiotic classes were approved over the subsequent 50 y. Recent efforts, including the U.S. National Action Plan for Combating Antibiotic-Resistant Bacteria, have contributed substantial resources to accelerate antibiotic development. Unfortunately, as highlighted by a 2021 report by the World Health Organization, gaps in the preclinical and clinical antibiotic pipelines remain, with most drugs in development being analogues of existing antibiotics (16).

The vast majority of antibiotics currently in use are microbial natural products or their derivatives. Historically, antibiotic development efforts focused on soil-based actinomycetes and involved screening of strains for zones of growth inhibition on petri dishes overlaid with various pathogens. As a result, actinomycetes are the source of many of the antibiotics used in modern medicine, including tetracyclines, aminoglycosides, macrolides, rifamycins, and vancomycin. Unfortunately, this untargeted approach to antibiotic development was labor intensive and yielded few candidate compounds relative to the number of strains screened, with identification of new antibiotic classes from actinomycetes becoming increasingly difficult over time. In the 1990s, antibiotic development efforts shifted largely to screening libraries of synthetic compounds for activity against defined bacterial targets. However, this approach proved largely unsuccessful, in part because of the challenge of identifying synthetic compounds that penetrate bacterial cells, and was ultimately abandoned by most pharmaceutical companies. The diminishing returns or failures of these previous antibiotic development platforms highlight the need for exploration of new sources of antibiotics and use of targeted approaches to identifying candidate compounds.

The human microbiota represents a vast and largely untapped source of novel antibiotics. The microbes that reside in and on our bodies have coevolved with humans and with each other, resulting in both symbiotic and antagonistic relationships among species occupying the same ecological niche. Among the cellular and molecular mechanisms underpinning these complex and bidirectional interactions is the production of antimicrobial compounds that specifically target competing species, including common human pathogens. The development of next-generation sequencing methods and improved tools for genomic and proteomic analysis enables a targeted and biologically informed approach to mining these interspecies relationships for novel antibiotics.

Stubbendieck and colleagues applied just such an approach to identify bacterial strains and a secreted protein with activity against *M. catarrhalis*. Using microbiome data generated from healthy children and children with upper respiratory symptoms, the authors identified a negative association between the relative abundance of *Rothia* and *M. catarrhalis* colonization. After demonstrating that specific *Rothia* strains inhibited *M. catarrhalis* growth at a distance and reasoning that the inhibitory factor was likely to be a protein, the authors further narrowed their search using comparative analyses of the genomes and secreted proteomes of these *Rothia* strains. This led to the identification of a putative peptidoglycan endopeptidase as the lead candidate for the observed activity against *M. catarrhalis*, with subsequent heterologous expression of a truncated form of this protein confirming its inhibition of *M. catarrhalis* growth. Critically, these *Rothia* strains inhibited three *Moraxella* species but not a panel of other Gammaproteobacteria, suggesting that the off-target effects of antimicrobial compounds secreted by these strains may be limited. However, further studies are needed to specifically determine the spectrum of activity of the endopeptidase SagA.

With the growing threat of antimicrobial resistance, other microbiome-based therapies are increasingly being explored as alternatives to antibiotics for infection prevention and treatment. A growing body of evidence indicates that commensal microbes in the upper respiratory tract influence the risk of colonization and infection by bacterial respiratory pathobionts (17, 18). The article by Stubbendieck and colleagues adds to this literature in supporting a role for *Rothia* species in colonization resistance to *M. catarrhalis*, although the presented microbiome analyses are limited by the small sample size and should be evaluated in other studies. Building upon prior observational studies, several recent clinical trials evaluated live bacteria for the elimination or prevention of respiratory pathobiont carriage. Deasy et al. reported that a single intranasal administration of a strain of *Neisseria lactamica* resulted in the eradication of and long-term protection from *N. meningitidis* colonization among university students (19). Similarly, Uehara et al. demonstrated that *S. aureus* colonization could be eradicated in most nasal carriers through intranasal administration of a *Corynebacterium* strain (20). These studies suggest that further elucidation of interspecies interactions within the human upper respiratory tract could lead to the development of nasal probiotics representing a new paradigm for respiratory infection prevention.

In conclusion, the human microbiota represents a largely unexplored source of novel biotherapeutics for infection prevention and treatment. The study by Stubbendieck and colleagues provides an example of how recent advances, including next-generation sequencing and improved methods for genomic and proteomic analyses, can accelerate development of rationally designed antibiotics. Such highly targeted approaches focused on the human microbiota could identify biotherapeutics that expand our toolset for combatting antibiotic resistance and for precisely engineering the microbiome for the promotion of human health.
